# Glyco-Dendrimers as Intradermal Anti-Tumor Vaccine Targeting Multiple Skin DC Subsets

**DOI:** 10.7150/thno.35059

**Published:** 2019-08-12

**Authors:** Sanne Duinkerken, Sophie K. Horrevorts, Hakan Kalay, Martino Ambrosini, Lisa Rutte, Tanja D. de Gruijl, Juan J. Garcia-Vallejo, Yvette van Kooyk

**Affiliations:** 1 Amsterdam UMC, Department of Molecular Cell Biology and Immunology, Cancer Center Amsterdam, Amsterdam Infection and Immunity Institute, Vrije Universiteit Amsterdam, Amsterdam, Netherlands; 2 Amsterdam UMC, Department of Medical Oncology, Cancer Center Amsterdam, Vrije Universiteit Amsterdam, Amsterdam, Netherlands

**Keywords:** DC-SIGN, Langerin, glyco-dendrimer, intradermal vaccination, tumor

## Abstract

The human skin is an attractive anti-tumor vaccination site due to the vast network of dendritic cell (DC) subsets that carry antigens to the draining lymph nodes and stimulate tumor specific CD4^+^ and CD8^+^ T cells in. Specific vaccine delivery to skin DC can be accomplished by targeting glycan coated antigens to C-type lectin receptors (CLRs) such as DC-SIGN expressed by human dermal DCs and Langerin expressed by Langerhans cells (LCs), which facilitate endocytosis and processing for antigen presentation and T cell activation. Although there are multiple human skin DC subsets, targeting individual DC subsets and receptors has been a focus in the past. However, the simultaneous targeting of multiple human skin DC subsets that mobilize the majority of the skin antigen presenting cells (APC) is preferred to accomplish more robust and efficient T cell stimulation. Dual CLR targeting using a single tumor vaccine has been difficult, as we previously showed Langerin to favor binding and uptake of monovalent glyco-peptides whereas DC-SIGN favors binding of larger multivalent glyco-particles such as glyco-liposomes.

**Methods:** We used branched polyamidoamine (PAMAM) dendrimers as scaffold for melanoma specific gp100 synthetic long peptides and the common DC-SIGN and Langerin ligand Lewis Y (Le^Y^), to create multivalent glyco-dendrimers with varying molecular weights for investigating dual DC-SIGN and Langerin targeting. Using DC-SIGN^+^ monocyte derived DC (moDC) and Langerin^+^ primary LC we investigated glyco-dendrimer CLR targeting properties and subsequent gp100 specific CD8^+^ T cell activation *in vitro*. *In situ* targeting ability to human dermal DC and LC through intradermal injection in a human skin explant model was elucidated.

**Results:** Dual DC-SIGN and Langerin binding was achieved using glyco-dendrimers of approximately 100kD, thereby fulfilling our criteria to simultaneously target LCs and CD1a^+^ and CD14^+^ dermal DC *in situ*. Both DC-SIGN and Langerin targeting by glyco-dendrimers resulted in enhanced internalization and gp100 specific CD8^+^ T cell activation.

**Conclusion:** We designed the first glyco-vaccine with dual CLR targeting properties, thereby reaching multiple human skin DC subsets *in situ* for improved anti-tumor CD8^+^ T cell responses.

## Introduction

Dendritic cells (DC) have a unique capacity to endocytose antigens and to activate naïve antigen- specific T-cells in the lymph nodes and thus are considered as the initiators of adaptive immune responses^1^, ^2^. Hence, DCs are widely explored for targeted anti-tumor immunotherapies. Efficient tumor elimination can be accomplished by the simultaneous induction of tumor specific CD4^+^ and CD8^+^ T cells 3. CD4^+^ T cells are activated via recognition of exogenous derived antigen loaded in major histocompatibility complex (MHC) class II, whereas CD8^+^ T cells via endogenous derived antigen loaded in MHC class I. For tumor cell killing anti-tumor immune responses rely on the induction of cytotoxic CD8^+^ T cells for which DCs need to shuttle endocytosed particles into the cross-presentation pathway to load tumor-derived epitopes into MHC class I 4. Various DC subsets have been described to have cross-presenting capacity *in vivo*, and are attractive targets to generate robust anti-tumor T cell immunity 4.

An important requirement for intracellular trafficking of antigens is the recognition of antigen by uptake receptors such as pathogen recognition receptors (PRR). A well-known group of PRRs is the C-type lectin receptor (CLR) family. CLRs have been extensively studied for their specific expression on DC subsets, their specificity of ligands, often carbohydrates, and their intracellular routing of antigen for loading on MHC class I and II for presentation to T cells 5. It is for this reason that CLRs have been used for vaccine delivery of (nano)particles to specific DC subsets using either antibody targeting or natural glycan ligands 5. Multiple CLRs showed potential in DC-targeted strategies inducing DC cross-presentation, e.g. DEC205, CLEC9A, the mannose receptor (MR) and dendritic cell-specific ICAM-grabbing non-integrin (DC-SIGN) 6-8. However, these receptors are often used to target a single DC subset, whereas targeting multiple subsets simultaneously may induce superior immune responses 9, 10.

*In vivo* vaccines are often applied in the human skin, since there is a high abundance of DCs and intradermal injections have shown to be dose-sparing compared to intramuscular delivery 11-13. Multiple DC subsets reside in the skin with LCs populating the epidermis and CD1a^+^, CD14^+^ and CD141^+^ DCs the dermis. CD1a^+^ dermal DC are inducers of cellular T cell responses, CD14^+^ dermal DC are better equipped to activate humoral responses and the CD141^+^ dermal DC subset is considered the most potent cross-presenting DC subset 14. As such, targeting multiple skin DC subsets simultaneously might elicit broader immune responses compared to single subset targeting. Though, it is becoming clear that the function of the different DC subsets can change depending on the vaccine format and mode of delivery 15. Especially specific CLR targeting can alter intracellular trafficking thereby influencing choice of CLR and DC subset targeting.

Although all subsets express multiple and partly overlapping CLRs, Langerin and DC-SIGN are two well-defined CLRs expressed by LCs and dermal DCs, respectively. Their glycan binding profile is partly overlapping as both recognize the Lewis type antigens, though DC-SIGN binds Lewis (Le) A,B , Y and X, whereas Langerin only recognizes Le^B^ and Le^Y^. Interestingly, both receptors show high affinity for Le^Y^, making this glycan an interesting candidate for dual CLR targeting 16. Furthermore, both receptors have been shown to efficiently deliver their cargo into the cross-presentation pathway, especially when combined with toll-like receptor (TLR) triggering 17, 18. TLRs are PRRs expressed by DCs that induce DC-mediated T cell activation via DC maturation and modulation of intracellular antigen trafficking in DCs 19. Combined triggering of TLR4 with DC-SIGN targeting resulted in cargo translocation to the cytosol, thus releasing it for proteasomal degradation and subsequent MHC I loading 17. For LCs combined targeting of Langerin and TLR3 using poly I:C enhanced cross-presentation and subsequent CD8^+^ T cell activation 18. Both DC-SIGN and Langerin are excellent targets for *in vivo* intradermal anti-tumor vaccination strategies.

The design of an off-the-shelve vaccine targeting multiple CLRs and skin DC subsets is difficult to accomplish via antibody targeting, however natural glycan ligands might be an option especially those that are shared by Langerin and DC-SIGN and display high affinity binding such as Le^Y^ 18, 19. Vaccine particle formulation can influence processing by DCs and subsequent adaptive immune responses 20-22. Spatial orientation of compounds and number of receptor ligands can change CLR binding and handling due to changes in avidity 23. Langerin and DC-SIGN appear to require different formulations to meet the needed combination of affinity and avidity for ligand endocytosis and cross-presentation. Indeed, our previous work demonstrated that relatively small sized glyco-peptides are targeted to Langerin (~3.5kD), whereas DC-SIGN preferentially binds large size glyco-particles (200nm) 16. This illustrates that a single glycan Le^Y^ structure may be used for Langerin targeting, whereas DC-SIGN may require multivalent presentation of Le^Y^, such as glyco-liposomes (200nm) to accomplish receptor mediated uptake, and the induction of cross-presentation 19. This different requirement to establish receptor-mediated uptake may be linked to functional differences of LCs and DCs to mediate viral and bacterial responses, respectively 24, 25. Moreover these DC subsets express a differential repertoire of TLR receptors to trigger maturation, such as viral TLR3 (Poly I:C) on LC and bacterial TLR4 (LPS) on dDC 26.

To meet the criteria for dual DC-SIGN and Langerin targeting, we explored different glyco-vaccine formulations which have the proper avidity for both receptors. To this end, we synthesized two multivalent Le^Y^ vaccines incorporating the CD4 and CD8 melanoma-specific gp100 antigen using two generations of well-defined, commercially available PAMAM dendrimer scaffolds which consist of branched subunits of amide and amine functionality 27. By creating glyco-dendrimers with either 4 or 32 functional groups containing the gp100 synthetic long peptide and Le^Y^ we elucidated dual targeting capacity and subsequent induction of cross-presentation. We describe generation 3 (G3) glyco-dendrimers simultaneously targeting DC-SIGN and Langerin, thereby enhancing gp100 specific CD8^+^ T cell activation when combined with a TLR stimulus. Furthermore, G3 glyco-dendrimers target both LCs and CD1a^+^ and CD14^+^ dDCs by which we have created the first glyco-vaccine targeting multiple human skin DC *in situ* for induction of anti-tumor immune responses.

## Results

### Multivalent Generation 3.0 glyco-dendrimers efficiently target both DC-SIGN and Langerin

**Figure 1:**
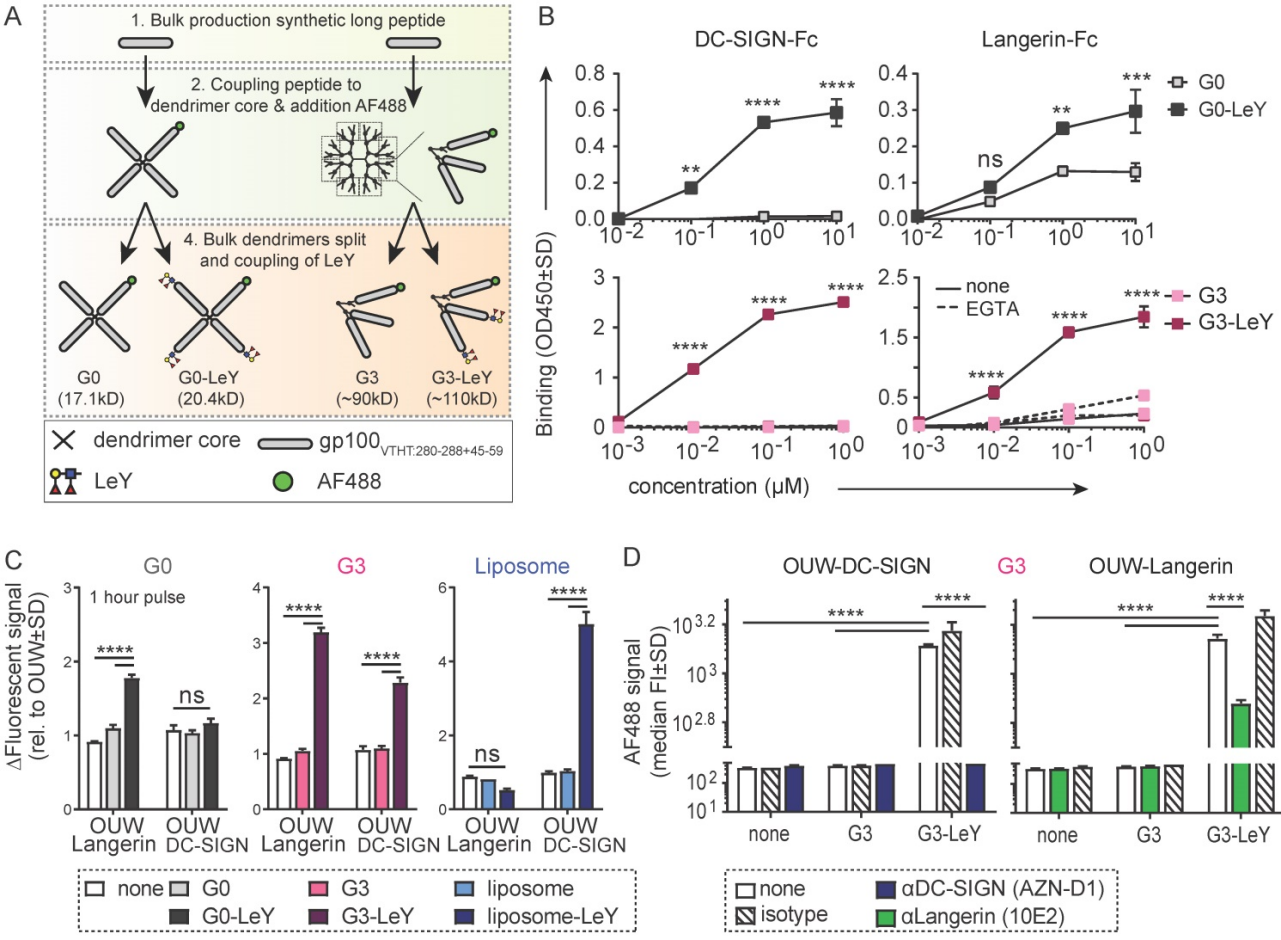
** Generation 3.0 (G3) glyco-dendrimers efficiently target both DC-SIGN and Langerin.** DC-SIGN and Langerin targeting was evaluated for different glyco-particles using CLR-Fc or a cell line transduced with DC-SIGN or Langerin** A.** Schematic representation of (glyco)-dendrimer synthesis. **B.** Binding of G0 (upper panels) or G3 (lower panels) (glyco)-dendrimers to human DC-SIGN-Fc (left panel) or Langerin-Fc (right panel) in the presence or absence of calcium depletion (EGTA) as measured by binding ELISA. **C.** Binding to membrane DC-SIGN and Langerin of different (glyco)-particles within 1 hour at 37 degrees; G0 (grey) or G3 (pink) (glyco)-dendrimers and (glyco)-liposomes (blue). **D.** DC-SIGN and Langerin specific binding by G3 glyco-dendrimers was evaluated using specific blocking antibodies for DC-SIGN and Langerin, or matched isotype control, prior to incubation. Data are representative of at least two independent experiments measured in triplicate ±SD (Statistical analysis: B two-way ANOVA Sidak's post hoc, C-D two-way ANOVA Tukey's post-hoc)

To design a glyco-vaccine that simultaneously targets DC-SIGN and Langerin, we generated two multivalent glyco-dendrimers differing in molecular weight, diameter and valency using the generation 0 (G0) or generation 3 (G3) PAMAM dendrimers, that have either 4 (G0) or 32 (G3) functional groups. As antigen, we included a synthetic long peptide of the melanoma epitope gp100 containing both HLA-DR4 CD4 and HLA-A02 CD8 restricted epitopes 17. Coupling of the gp100 peptide to the G0 or G3 dendrimers (Figure **S1A**) resulted in antigen specific multivalent dendrimers of 16.4kD (G0) (**Figure S1B**) and approximately 52nm (G3) (**Figure S1C**). Dendrimers were further modified with AF488 for tracking purposes and the targeting glycan Le^Y^ 16, thereby creating multivalent fluorescent glyco-dendrimers (schematic representation **Figure S1A**). Fluorescent labeling was done such that coupling ensured equal fluorescence and epitope content of total molecules between non-glycosylated and glyco-dendrimers (schematic representation **Figure 1A**). Using DC-SIGN- and Langerin-Fc we confirmed recognition of both G0 and G3 glyco-dendrimers by the carbohydrate recognition domain (CRD) of both receptors, whereas the non-glycosylated dendrimers were not recognized by either soluble receptor in an ELISA detection system (**Figure 1B**). Further, calcium-dependent binding of the dendrimers to DC-SIGN- and Langerin-Fc was confirmed using the calcium chelator EGTA. To verify whether the membrane organization of DC-SIGN and Langerin, that cluster in tetra- and trimers in the membrane respectively, may influence the binding and internalization of the different glyco-dendrimers, we made use of an OUW cell-line transduced with DC-SIGN or Langerin. After one hour pulse the smaller G0 glyco-dendrimers did not bind DC-SIGN-expressing OUW cells, but did bind to Langerin-expressing OUW cells (**Figure 1C**, *left panel*). This is in keeping with our previous findings that in contrast to DC-SIGN, Langerin has a preference for binding smaller molecules 16. Interestingly, the G3 glyco-dendrimers could efficiently target both DC-SIGN and Langerin after one hour incubation (**Figure 1C**, *middle panel*). Glyco-liposomes served as a positive control for DC-SIGN and, as expected, they solely bound DC-SIGN and not Langerin as previously shown (**Figure 1C**, *right panel*). Using blocking antibodies specific for either DC-SIGN or Langerin, we could confirm specific binding of G3 glyco-dendrimers to both receptors (**Figure 1D**). We therefore concluded that G3 glyco-dendrimers have all the requirements to serve as skin multi-DC subset targeting glyco-vaccine via its binding to both Langerin and DC-SIGN.

### Enhanced uptake of glyco-dendrimers by moDC and primary LC mediated via DC-SIGN and Langerin

**Figure 2:**
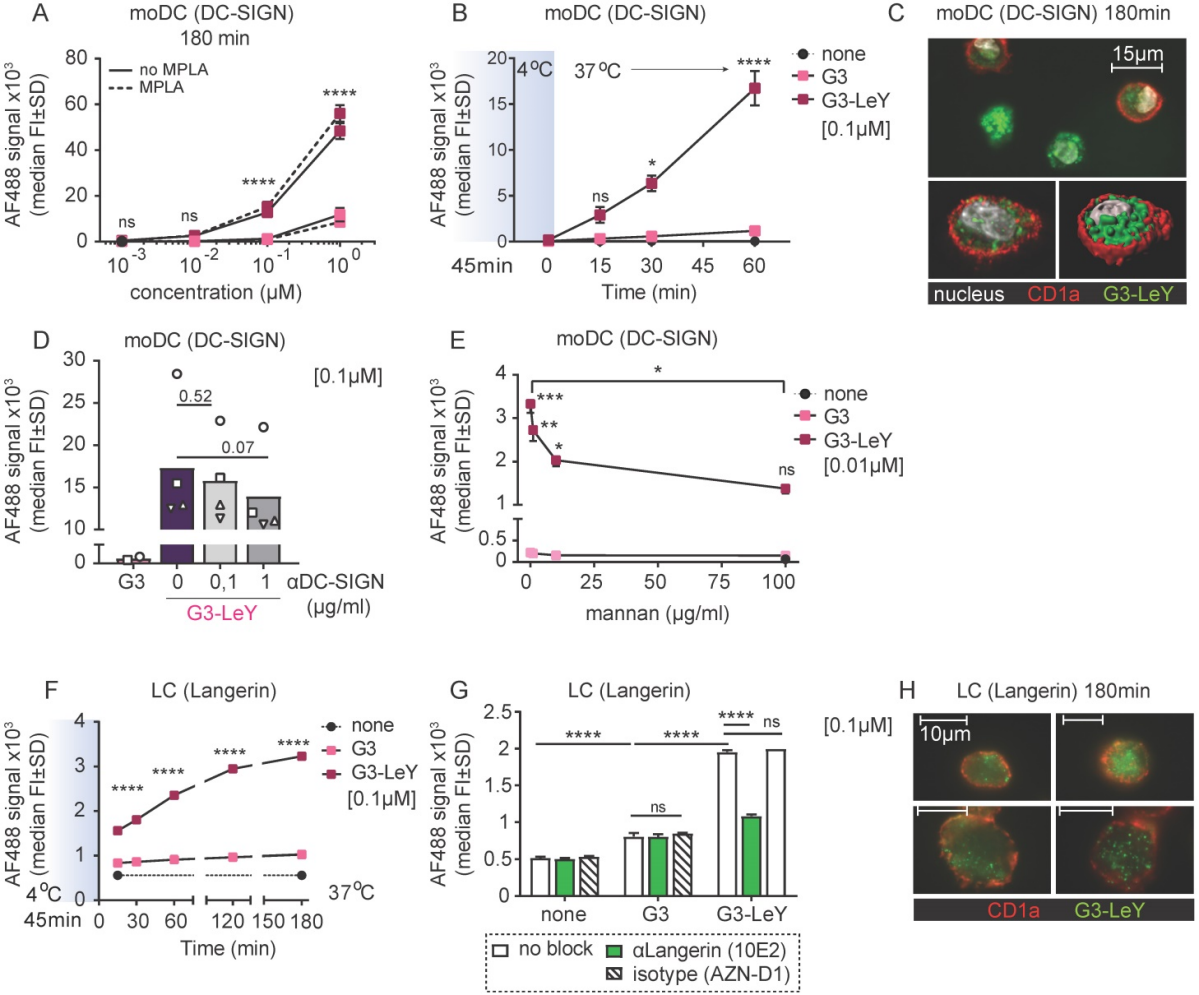
** Enhanced glyco-dendrimer binding and uptake by moDC via DC-SIGN and primary LC via Langerin.** Binding and uptake of G3 (glyco)-dendrimers was evaluated for DC-SIGN^+^ moDC and Langerin^+^ primary LCs. **A.** Dose-response following a 3 hour pulse, wash and 45 minutes chase of moDC with G3 (glyco)-dendrimers in the presence (dotted line) or absence (solid line) of TLR4 stimulus MPLA. Representative of n=3 measured in triplicate ±SD** B.** Binding and uptake of (glyco)-dendrimers over-time by moDC following a 45 minutes pulse (no wash) at 4^o^C. n=2, ±SD **C.** Imaging microscopy of moDC following 3 hour incubation at 37^ o^C with glyco-dendrimers (green). Membrane was stained using anti-CD1a (red) and nucleus using DAPI (white)** D-E.** Involvement of DC-SIGN in binding and uptake of G3 (glyco)- dendrimers was evaluated using a 3 hour pre-incubation with anti-DC-SIGN (C) or 30 minutes pre-incubation with the natural ligand mannan (D) followed by 1 hour incubation with (glyco)-dendrimers. (C) n=4, each symbol represents a donor, (D) representative of n=2 measured in triplicate ±SD **F**. Binding and uptake of (glyco)-dendrimers over-time by primary LC following a 45 minutes pulse (no wash) on 4^o^C. Representative of n=2 measured in triplicate **G.** Langerin involvement in binding and uptake of (glyco)-dendrimers by primary LCs was evaluated using 30 minutes pre-incubation an anti-Langerin blocking antibody followed by 1 hour co-incubation with (glyco)-dendrimers. Representative of n=3 measured in triplicate ±SD **H.** Imaging microscopy of primary LC following 3 hours incubation at 37^ o^C with glyco-dendrimers (green). Membrane was stained using anti-CD1a (red) (Statistical analysis: A,E two-way ANOVA Sidak's post hoc; B,D,F two-way ANOVA Tukey's post hoc; C one-way ANOVA Dunnett's post hoc)

To determine whether the uptake of G3 glyco-dendrimers by moDCs and primary LCs is also mediated by Langerin and DC-SIGN, we compared uptake of glyco-dendrimers to that of non-glycosylated dendrimers. Glyco-dendrimers are efficiently taken up by moDCs as compared to non-glycosylated dendrimers within 3 hours at 37^ o^C, and a targeting effect is already evident at low concentrations (**Figure 2A**). To elucidate whether glyco-dendrimers show increased binding and uptake over time compared to non-glycosylated dendrimers at constant exposure, we pre-incubated moDC at 4^o^C with the respective dendrimers to ensure receptor binding, followed by direct incubation up to one hour at 37^o^C. We observed a clear increase of AF488 signal from glyco-dendrimers over-time as compared to non-glycosylated dendrimers, indicating that glyco-dendrimers are rapidly bound and internalized by moDC (**Figure 2B**). Imaging microscopy confirmed internalization of the glyco-dendrimers by moDC following 3 hours incubation at 37^ o^C (**Figure 2C**). Involvement of DC-SIGN in enhanced uptake by moDC was elucidated using an anti-DC-SIGN antibody known to bind the CRD and induce internalization of DC-SIGN. Prior to incubation with dendrimers, moDC were incubated with anti-DC-SIGN at 37^o^C to ensure receptor occupation and partial internalization. Since DC-SIGN is a non-recycling receptor, internalized DC-SIGN will no longer be available for ligand binding. Interestingly, targeting of glyco-dendrimers to DC-SIGN appeared to be very efficient since pre-incubation with anti-DC-SIGN for 30 minutes did not affect binding and uptake, which for glyco-liposomes was sufficient (**Figure S2A**). To demonstrate DC-SIGN-mediated internalization of glyco-dendrimers, moDC had to be pre-incubated for at least 3 hours using the blocking antibody (**Figure 2D**). Remarkably, a clear blocking effect of glyco-dendrimer uptake was seen when moDC were pre-incubated with increasing concentrations of the high affinity ligand of DC-SIGN, mannan, confirming that DC-SIGN is indeed responsible for the enhanced binding and uptake of glyco-dendrimers by moDC (**Figure 2E**).

To elucidate whether our compound also targeted Langerin on primary LCs, we used LCs obtained by two day emigration from epidermal sheets. Langerin expression is lower in emigrated LC compared to steady-state LC, nevertheless, expression levels were sufficient to elucidate Langerin targeting (**Figure S2B**). To track G3 glyco-dendrimer binding and uptake over time, primary LC were pre-incubated at 4^o^C for 45 minutes, followed by incubation at 37^o^C for multiple time-points. Similar as for moDC, we found increased signal for glyco-dendrimers compared to non-glycosylated dendrimers already at 15 minutes, indicating rapid binding and uptake by primary LCs as confirmed by imaging microscopy (**Figure 2F,H**). For primary LCs, pre-incubation with an anti-Langerin blocking antibody resulted in an almost complete abrogation of enhanced glyco- dendrimer uptake (**Figure 2G**), confirming the targeting ability of G3 glyco-dendrimers to Langerin.

### Enhanced uptake of glyco-dendrimers by DC-SIGN^+^ and Langerin^+^ human skin DCs *in situ*

**Figure 3:**
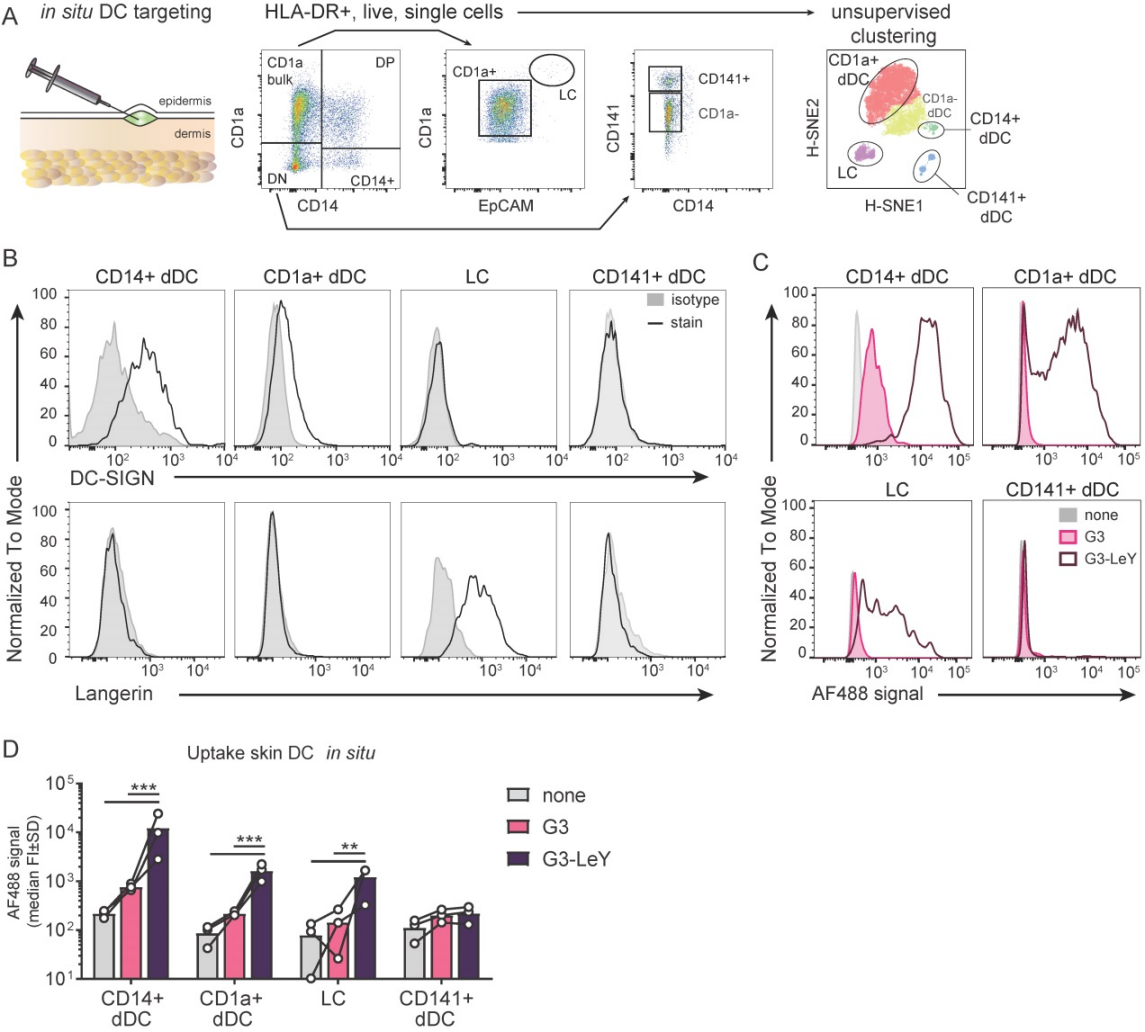
** Glyco-dendrimers target multiple skin DC subsets for enhanced uptake. A.** Gating strategy of human skin DC subsets following injection and two day emigration **B.** DC-SIGN and Langerin surface expression on two day emigrated skin DC subsets **C-D.** Binding and uptake of (glyco)-dendrimers by human skin DC subsets following *in situ* injection. n=3 plus representative histograms. Each dot represents a donor. (Statistical analysis: two-way ANOVA Dunnett's post hoc)

Since we confirmed Langerin and DC-SIGN mediated targeting and uptake specificity by G3 glyco-dendrimers, we set out to explore whether this specificity remained when injected in human skin that harbors all the different human skin DC subsets expressing DC-SIGN or Langerin. We used a human skin explant model 28 to inject the G3 (glyco)- dendrimers and verify targeting to human skin DCs expressing DC-SIGN or Langerin. This model represents a steady-state environment with the physiological localization, phenotype and ratio of the different human skin DC subsets. As such, it supplies the best possible representation of the human skin to study specific DC targeting by the glyco-vaccine upon intradermal delivery. Following injection, cells were allowed to emigrate for two days and analyzed by FACS. Skin APC subsets were defined based on HLA-DR and subset-specific markers CD1a, CD14, CD141 and EpCAM (**Figure 3A**) using manual gating and unsupervised clustering to confirm number of subsets. DC-SIGN and Langerin expression was evaluated after two day skin DC emigration for all subsets. In concordance with literature we found Langerin on LCs and DC-SIGN expressed by CD14^+^ dDC and to a very small extent by CD1a^+^ dDC (**Figure 3B**). As expected, CD14^+^ dDC and LC showed higher uptake of G3 glyco-dendrimers compared to the non-glycosylated dendrimers. Interestingly, we found CD1a^+^ dDC to also efficiently take up the glyco-dendrimers (**Figure 3C-D**) despite their low expression levels of DC-SIGN (**Figure 3B**), suggesting G3 glyco-dendrimers can already efficiently target DC-SIGN at lower expression levels. For the Langerin^-^ and DC-SIGN^-^ CD141^+^ dDCs we did not find any enhanced uptake of the glyco-dendrimers (**Figure 3C-D**). Altogether, these data show the ability of G3 glyco-dendrimers to efficiently target multiple skin DC subsets *in situ*.

### G3 glyco-dendrimers enhance cross-presentation for tumor specific CD8^+^ T cell activation

**Figure 4:**
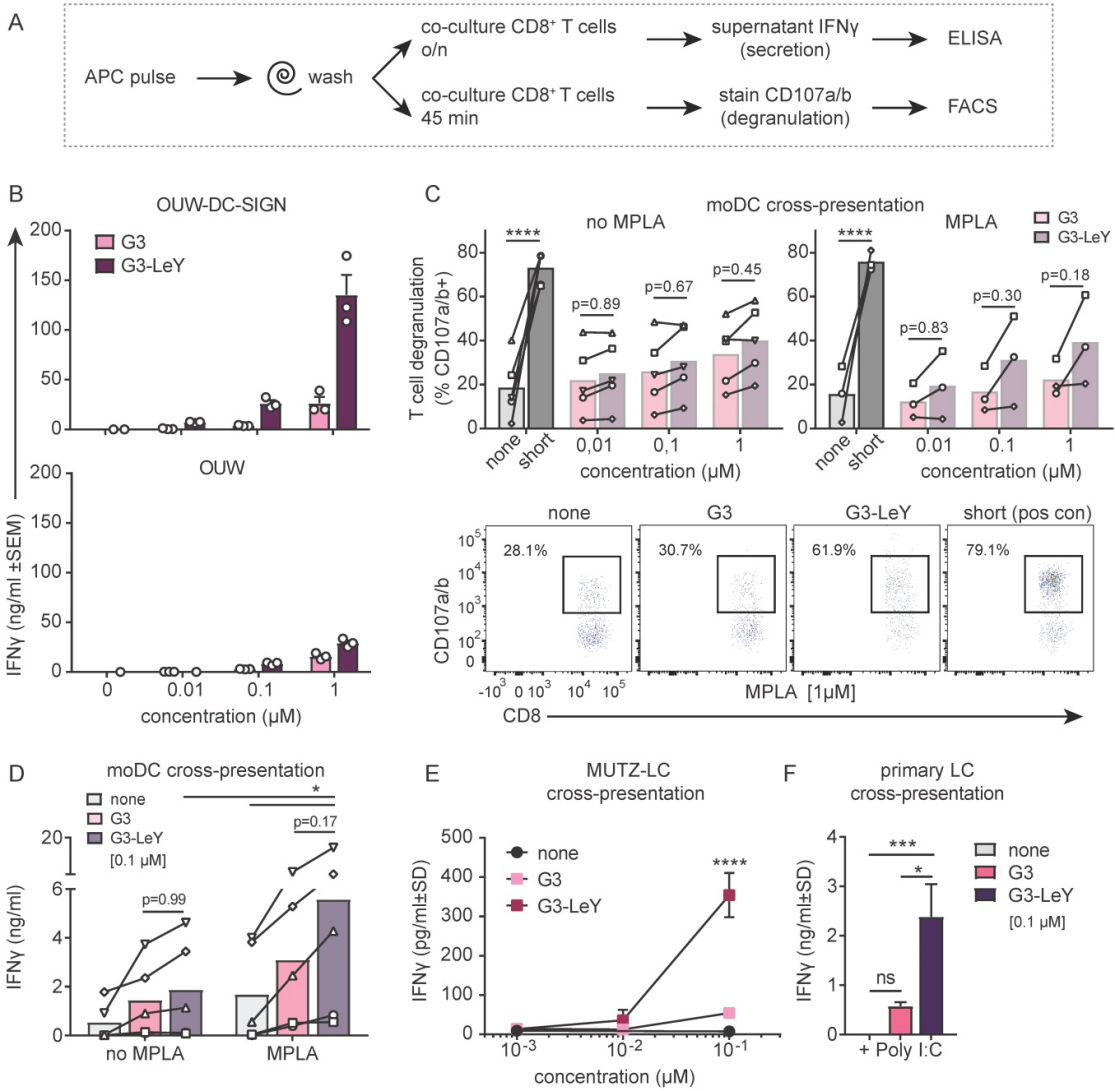
** Enhanced cross-presentation of glyco-dendrimers by moDC and primary LC. A.** APC were pulsed with the (glyco)-dendrimers for 3 hours or 30 minutes, washed and co-cultured with a gp100 specific T cell clone for either 45minutes or o/n. CD8^+^ T cell activation was measured by FACS using CD107a/b staining for degranulation or by ELISA for IFNγ secretion in the supernatant **B.** IFNγ production of gp100 specific T cells after o/n culture with pulsed OUW cells transduced with DC-SIGN (upper panel) or Langerin (lower panel).Representative of n=2±SD measured in triplicate** C.** Degranulation of gp100 specific T cells following 45min culture with 3 hour pulsed moDC in the absence (left) or presence (right) of the TLR4 stimulus MPLA. n=3-5, each symbol represents a donor. Representative dot plots for MPLA 1µM **D.** IFNγ production by gp100 specific T cells following o/n culture with 30 minutes pulsed moDC in the absence or presence of the TLR4 stimulus MPLA. n=5, each symbol represents a donor. **E-F.** IFNγ production by gp100 specific T cells following o/n co-culture with 3 hour pulsed (E) MUTZ-LC or (F) primary LC in the presence of TLR3 stimulus Poly I:C. Representative of n=2±SD measured in triplicate. (Statistical analysis: C,D two-way ANOVA Sidak's post hoc, D,E one-way ANOVA Tukey's post hoc)

To ensure proper delivery for CD4^+^ and CD8^+^ T cell activation, we used gp100 specific T cell clones recognizing either the CD4 or CD8 minimal epitope. First, we verified activation of CD4^+^ T cells following overnight co-culture with moDC. We found gp100 specific CD4^+^ T cell activation as measured by IFNγ secretion, which increased upon glyco-dendrimer pulse both in the absence and presence of TLR4 stimulation (MPLA) (**Figure S3**). To verify whether targeting and enhanced uptake of the G3 glyco-dendrimers to both DC-SIGN and Langerin results in enhanced CD8^+^ T cell activation, we first verified cross-presentation via DC-SIGN using a DC-SIGN expressing OUW cell line and DC-SIGN^+^ moDC. Briefly, APCs exposed to (glyco)-dendrimers were co-cultured with a gp100 specific T cell clone recognizing the gp100 HLA-A2 minimal epitope and degranulation or IFNγ secretion were subsequently measured (**Figure 4A**). We observed that the G3 glyco-dendrimers induced CD8^+^ T cell activation by DC-SIGN^+^ OUW cells as measured by increased IFNγ secretion, without addition of a TLR stimulus (**Figure 4B**, *upper panel*). For moDC we have previously shown that TLR4 signaling alters routing for DC-SIGN targeted vaccines inducing enhanced CD8^+^ T cell activation compared to untargeted vaccines 17. To elucidate the influence of TLR4 signaling on cross-presentation of the glyco-dendrimers compared to non-glycosylated dendrimers, we pulsed moDC in the presence or absence of the TLR4 stimulus MPLA followed by a direct short co-culture to avoid influence of MPLA induced moDC maturation. MoDC show enhanced degranulation of gp100 specific CD8^+^ T cells already after a short co-culture following combined triggering of TLR4 (MPLA) with G3 glyco-dendrimer targeting compared to non-glycosylated dendrimers (**Figure 4C**). In concordance, overnight co-culture of gp100 T cells with glyco-dendrimer pulsed moDC in the presence of MPLA enhanced IFNγ production compared to non-glycosylated dendrimers, but also overall IFNγ production (**Figure 4D**). This indicates DC-SIGN expressed by moDC efficiently routes antigens into the cross-presentation pathway only under the influence of TLR4 signaling, underlining the need for the presence of a potent adjuvant in the vaccine formulation.

Next, we verified whether the enhanced G3 glyco-dendrimer targeting to Langerin also resulted in antigen cross-presentation and enhanced CD8^+^ T cell activation. LC derived from MUTZ cells induced increased IFNγ production by gp100 specific CD8^+^ T cells when pulsed with glyco-dendrimers compared to non-glycosylated dendrimers (**Figure 4D**). As primary LCs are the *in vivo* target, we isolated primary LCs from human epidermal sheets to elucidate cross-presentation of G3 glyco-dendrimers in combination with the TLR3 stimulus Poly I:C, known to enhance primary LC induced CD8^+^ T cell activation 18. There was increased IFNγ production by gp100 specific CD8^+^ T cells following o/n culture with G3 glyco-dendrimer-pulsed primary LCs compared to primary LCs pulsed with non-glycosylated dendrimers (**Figure 4E**). This indicates Langerin targeting by G3 glyco-dendrimers on primary LCs routes antigens into the cross-presentation pathway for enhanced CD8^+^ T cell activation. Overall, these data show that the enhanced binding and uptake of G3 glyco-dendrimers by DC via DC-SIGN or Langerin, combined with a TLR stimulus, induces an increase in degranulation and IFNγ production by gp100 specific CD8^+^ T cells.

## Discussion

Here, we successfully designed a gp100 melanoma-specific vaccine that targets multiple skin DC subsets through its dual specificity for DC-SIGN and Langerin receptors. We show that the molecular architecture of the vaccine is essential in enabling efficient dual targeting, as conditions for targeting need to be met for two different receptors. Generation 3.0 glyco-dendrimers have the ability to target both DC-SIGN and Langerin through binding the Le^Y^ glycan, thereby reaching LC, CD1a^+^ and CD14^+^ dDC within the human skin *in situ*. Combination of glyco-dendrimer targeting to DC-SIGN and Langerin with a TLR stimulus resulted in cross-presentation and enhanced gp100 specific CD8^+^ T cell activation, illustrating their great potential for intradermal anti-tumor vaccination strategies and shedding additional light on the requirements for glyco-vaccine formulation.

Although DC-SIGN and Langerin have overlapping glycan binding specificity, their internalization and intracellular processing differs greatly. Despite their difference in intracellular compartmentalization, different studies showed that both receptors process antigen intracellularly for cross-presentation. However, our earlier work showed induction of cross-presentation by Langerin via small glyco- peptides, whereas by DC-SIGN via large glyco- liposomes 16. Interestingly, often viruses and bacteria use DC-SIGN to either escape the immune system or alter T helper cell responses via intracellular signaling cascades 29. In contrast, Langerin- mediated uptake of viruses such as HIV and fungi mediates clearance 30, which might be due to the specific formation of Birbeck Granules upon Langerin-mediated internalization. Birbeck Granules are subdomains of the endosomal recycling compartment 31 that regulate antigen degradation differently from other endocytic compartments. 32, 33. These discrepancies might explain why until now no glyco-vaccine has been developed with the capacity to target both receptors simultaneously whilst inducing cross-presentation.

Interestingly, DC-SIGN can also be efficiently targeted for *in vivo* cross-presentation via Le^X^ coupled glyco-dendrimers with increasing multivalency, a technique not yet explored for Langerin targeting 34. In this study, we used PAMAM dendrimer scaffolds to create two formulations of melanoma-specific glyco-dendrimers that differed in molecular weight to investigate targeting properties to both DC-SIGN and Langerin. The smallest G0 glyco-dendrimers with four functional groups only showed targeting ability towards Langerin and not DC-SIGN, whereas higher multivalency G3 glyco-dendrimers harboring 32 functional groups showed binding to both receptors on the cellular membrane. Targeting with G3 glyco-dendrimers resulted in enhanced binding and uptake for both moDC and primary LC over time. Interestingly, low generation PAMAM dendrimers have high flexibility 27, 35 and efficient binding to Langerin and DC-SIGN may require membrane movement of the CLRs explaining enhanced binding at 37°C.

Membrane organization of DC-SIGN can affect glycan-coated ligand binding, as shown for synthetic hyperbranched polymers containing mannose, but also viral entry 36, 37. Interestingly, our results show that the same targeting moiety on different formulations of cargo can alter targeting properties to a single receptor. Using previously described glyco- liposomes known to target DC-SIGN 16 we found efficient blocking when moDC were simultaneously incubated with the common blocking antibody AZN-D1. Strikingly, for G3 glyco-dendrimers the strong binding to moDC showed partial DC-SIGN blocking with the AZN-D1 antibody, whereas pre-incubation with the high affinity natural binding ligand mannan 38 efficiently blocked the binding and uptake of glyco-dendrimers by DC-SIGN^+^ moDC, indicating high binding efficiency of G3 glyco-dendrimers to DC-SIGN. Although mannan can also bind to other receptors such as the mannose receptor (MR), our glycan of choice (Le^Y^) is high affinity for DC-SIGN and thus binding to other receptors on DC is unlikely.^39^

Glyco-dendrimer targeting to primary LC was considerably lower compared to moDC, possibly due to lower expression levels of Langerin. MoDC have high expression of DC-SIGN, whereas primary LCs, migrated from the epidermis for two days, show decreased Langerin expression levels (**Figure S2B**). The phagocytic capacity of the different cell types can also be important for *in situ* targeting, since DC-SIGN^high^ CD14^+^ dDC showed greater glyco- dendrimer uptake compared to DC-SIGN^low^ CD1a^+^ dDC and epidermal LCs. CD14^+^ dDC are considered to have a monocytic lineage background 40 and hence may have higher phagocytic capacity compared to other skin DCs, as represented by moDC.

Anti-tumor vaccination strategies aim to induce cross-presentation for the priming of tumor-specific CD8^+^ T cells and effective tumor cell killing. In murine models, XCR1^+^ DCs, present in lymph nodes and peripheral tissues, have been postulated as potent target candidates for induction of cytotoxic T cell responses 41 and anti-tumor immune responses 42. The dermis of human skin contains CD141^+^ cross-presenting DCs, which are considered to be the homologue of murine CD103^+^XCR1^+^ dermal DCs 43. Nonetheless, we did not observe *in situ* targeting towards CD141^+^ dDCs by the G3 glyco-dendrimers, consistent with their lack of DC-SIGN or Langerin expression. Although this skin DC subset is considered a potent target candidate for induction of cytotoxic anti-tumor immune responses 44, it represents a minority of the total dermal DC pool. In contrast, we show efficient G3 glyco-dendrimer targeting towards LCs and the larger pool of CD1a^+^ and CD14^+^ dDCs. Targeting of LCs and CD14^+^ dDCs via Langerin and DC-SIGN, respectively, can induce cross-presentation when different glyco-vaccines are used 8, 18, 28. Here we show that G3 glyco-dendrimers are cross-presented via both DC-SIGN and Langerin when combined with a TLR stimulus, thereby enhancing gp100 specific CD8^+^ T cell activation. Combining CLR targeting with TLR stimuli can induce cross-talk and alter the intracellular fate of CLR bound cargo. We previously showed that simultaneous TLR4 and DC-SIGN triggering translocated DC-SIGN cargo into the cross-presentation pathway, most likely via endosomal escape and proteasomal degradation 17. Also combined activation of Langerin and TLR3 enhanced CD8^+^ T cell activation 18, but a direct link between Langerin and TLR3 signaling for cross-presentation has not yet been demonstrated.

Combination of glycan CLR targeting and TLR stimulation is gaining interest in the vaccination field as it can elicit superior humoral and cellular immunity when incorporated within a single vaccine particle 45, 46. Our dual CLR targeting G3 glyco-dendrimers have the potential for adjuvant coupling, which is an interesting feature for future research into glyco-adjuvant vaccines for intradermal anti-tumor vaccination strategies. Furthermore, recent advances in anti-tumor immunotherapies show the importance of neo-antigens and CD4^+^ T cell help for induction of long lasting anti-tumor CD8^+^ T cell immunity 47, 48. The high branched multivalent G3 glyco-dendrimers allow inclusion of multiple TAA, such as neo-antigens and combined CD4 and CD8 restricted epitopes, thereby generating a highly diverse vaccine platform. Here we already combined the melanoma specific gp100 HLA-DR4 and HLA-A2 restricted epitopes for activation of both CD4^+^ and CD8^+^ gp100 specific T cells (**Figure S3 and figure 4**).

Evaluating the therapeutic efficacy and immune responses induced upon multiple DC subset targeting of our newly designed glyco-vaccine would be of great importance. However, since we specifically designed a glyco-vaccine for dual targeting of DC- SIGN and Langerin on human skin DC, a murine model with comparable expression by similar DC subsets is imperative. Although the murine CD209a/ SIGNR5, or mDC-SIGN, displays some similarities to the human counterpart 49 the glycan binding profile has not been studied. As such, a humanized DC-SIGN murine model would be needed to evaluate *in vivo* efficacy of our glyco-vaccine specifically designed to target human DC-SIGN. While a humanized DC-SIGN murine model is available, it harbors DC-SIGN expression under the CD11c promotor thereby containing multiple hDC-SIGN+ DC subsets 50. Unfortunately, this renders the model unsuitable for the glyco-vaccine of this study since human skin has restricted DC-SIGN expression to CD14^+^ and CD1a^+^ dermal DCs (**Figure 3B**).

In summary, we designed an intradermal glyco-vaccine simultaneously targeting multiple human skin DC subsets *in situ*. Simultaneous targeting was accomplished by the use of G3 glyco- dendrimers targeting both DC-SIGN and Langerin, which enhanced activation of gp100 specific CD8^+^ T cells in combination with TLR stimulation. These promising results pave the way for future studies investigating the *in vivo* behavior of G3 glyco- dendrimers following intradermal vaccination and induction of systemic anti-tumor immune responses via dual DC-SIGN and Langerin targeting.

## Material and Methods

### Cells

OUW, OUW-DC-SIGN and OUW-Langerin B cell lines were cultured in RPMI (Invitrogen, USA) supplemented with 10% FCS (Lonza), 50U/ml penicillin, 50ug/ml streptomycin, 2mM glutamine (all BioWhittaker, USA) (complete RMPI).

Monocytes were isolated from buffy coats (Sanquin, The Netherlands) using serial Ficoll/Percoll gradient centrifugation and cultured in complete RPMI. For human moDC differentiation and DC-SIGN expression, rhGM-CSF plus rhIL-4 (500U/ml; Biosource, Belgium) were added for 4-6 days.

Primary LCs were isolated from human skin explants (Bergman Clinics, Bilthoven, The Netherlands) obtained within 24 hours following abdominal resection of healthy donors with informed consent. Part of the epidermal and dermal sheet (5-mm thickness) were removed using a dermatome blade (Zimmer, Germany), rinsed with PBS plus gentamycin (10µg/ml; Lonza) and incubated in serum free IMDM supplemented with 50U/ml penicillin, 50ug/ml streptomycin, 2mM glutamine, gentamycin and dispase II (1mg/ml, Roche Diagnostics) for 2 hours at 37^o^C. The epidermal sheet was separated from the dermis using tweezers, followed by two day culture in IMDM supplemented with 10% FCS, penicillin, streptomycin, glutamin, gentamycin (complete IMDM skin medium) and rhGM-CSF (500U/ml) for LC migration at 37^o^C. LCs were harvested and purified using a Ficoll gradient (>85%). For purity evaluation LCs were incubated with anti-human antibodies against HLA-DR (clone G46-6, BD Biosciences), CD1a (clone HI149, BD Biosciences) and Langerin (clone 10E2, Biolegend).

MUTZ-LC were kindly provided by Prof. dr. S. Gibbs and cultured as previously described 51. Cells were used when >70% were CD1a and Langerin positive.

The retroviral TCRαβ transduced T cell clone specific for the gp100_280-288_ HLA-A2 minimal epitope (YLEPGPVTA) 52 and HLA-DRB1*0401-restricted T cell line Bridge gp:44 B8 53 were cultured in IMDM medium (Invitrogen), supplemented with Yssel's medium (20 μg/ml human transferrin (Boehringer), 5 μg/ml insulin (Sigma-Aldrich), 2 μg/ml linoleic acid (Calbiochem), 2 μg/ml palmitic acid (Calbiochem), 0.25% BSA (Sigma-Aldrich), and 1.8 μg/ml 20-amino ethanol (Sigma-Aldrich)), 1% human serum (Sigma- Aldrich) and penicillin, streptomycin, glutamin. Cells were expanded for 10-12 days in the presence of IL-2 (100IU/ml; Peprotech) and PHA-L (2µg/ml; Sigma) prior to storage in liquid nitrogen. For co-cultures T cells were either used at day 12 in expansion or thawed and rested for at least 6 hours before co-culture.

### FACS staining, measurement and analysis

A pre-mix of surface marker antibodies diluted in PBS plus 0,5% BSA (0,5% PBA; Roche) was prepared prior to incubation for 30 minutes on ice. Unbound antibodies were washed away with PBS, followed by fixation using 4% paraformaldehyde (PFA; Electron Microscopy Science) for 20 minutes on ice. Next, cells were washed two times with PBS and resuspended in 0,5% PBA for measurement using the FACS Fortessa-X20 (BD). Analysis was performed with FlowJo 10 software (Tree Star, Ashland, OR, USA).

### Peptide synthesis

Thz-VTHTYLEPGPVTANRQLYPEWTEAQRLD-(Abu)_3_-C peptide was synthesized at the GlycO2pep unit at our lab by microwave assisted solid phase peptide synthesis using Fmoc chemistry on a peptide synthesizer (Liberty blue peptide synthesizer, CEM). The peptide was deprotected with 92.5% TFA, 2.5% MilliQ, 2.5% TIS and 2,5% EDT cleavage solution. After collection, the peptide was lyophilized and purified on a preparative Ultimate 3000 HPLC system (Thermo Fisher) over a Vydac 218MS1022 C18 25x250mm column (Grace Vydac). Mass and purity were confirmed by UHPLC-MS on a Ultimate 3000 UHPLC system (Thermo Fisher) hyphenated with a LCQ-Deca XP Iontrap ESI mass spectrometer (Thermo Finnigan) using a RSLC 120 C18 Acclaim 2.2um particle 2.1 x 250 mm column and ionizing the sample in positive mode.

### Glyco-dendrimer synthesis

G0/G3-Gp100-AF488-LewisY constructs were synthesized via thiol-ene mediated reactions. In short, PAMAM generation 0 or 3 dendrimer (Sigma) were functionalized with maleimide or LC-SMCC bifunctional crosslinker (ThermoFisher), respectively. After purification, the dendrimer was loaded with GP100 long synthetic peptide Thz-VTHTYLEPGPVTANRQLYPEWTEAQRLD-(Abu)_3_-C through its C- terminal cysteine. After removing the excess peptide, labelling and glycation was achieved by unmasking the N-terminal thioproline (Thz; Novabiochem) and reacting it with AF488 (Invitrogen) /Lewis Y (Elicityl) pentasaccharide maleimide. G0 dendrimer MW was determined using mass spectrometry. Particle size of G3-PAMAM-GP100, dissolved in MilliQ at 0.1 mg/ml and 0.05 mg/ml, was determent using a dynamic and static light scattering measurement (Malvern Zetasizer Nano S, Breda, Netherlands). The average of 3 measures was used to calculate the particle size. These measurements indicate that the average particle size of our peptidic dendrimer is 52.03 nm.

### Binding ELISA DC-SIGN-Fc and Langerin-Fc

DC-SIGN-Fc and Langerin-Fc were obtained as previously described 54. Dendrimers with and without LeY conjugation were coated on NUNC maxisorb plates (Roskilde) o/n at 4^o^C. Following removal of free dendrimers with TSM for DC-SIFN-Fc or HBSS (Invitrogen) for Langerin-Fc wash, wells were blocked using 1% BSA (Fraction V, Fatty acid free, PAA laboratories) in TSM or HBSS. Next, dendrimers were incubated with 2µg/ml DC-SIGN-Fc or Langerin-Fc diluted in TSM or HBSS plus 0,5% BSA, respectively, for 2 hours at RT. After 3 washes, binding was detected using a HRP-labeled F(ab')2 goat anti-human IgG specific antibody. HRP binding was visualized using 3,3',5,5'-tetramethylbenzidine (TMB) substrate (Sigma Aldrich) followed by measurement at 450nm.

### Binding and uptake assays

Triplicates of 5x10^4^ cells were plated in a 96-U bottom plate (Greiner) and incubated with AF488 conjugated (glyco)-dendrimers or vehicle control (max. 0,2% DMSO) diluted in serum free IMDM for LCs and complete RPMI for moDCs. When indicated cells were pre-incubated for 1 hour at 4^o^C, followed by incubation at 37^o^C for indicated time-points. Next, cells were stained with a fixable viability dye eFluor780 (FVD; eBioscience), anti-human HLA-DR BV510, CD1a APC (clone HI149; BD) (moDC) and EpCAM BV421 (clone EBA-1; Biolegend) (LCs). Binding and uptake was analyzed using FACS.

For antibody blocking assays cells were pre-incubated with 20µg/ml mouse-anti-human IgG1 Langerin (10E2) or DC-SIGN (AZN-D1) for 30 minutes at 37^o^C, followed by addition of (glyco)-dendrimers for 1 hour at 37^o^C with blocking antibodies at a final concentration of 10µg/ml. For 3 hour pre-incubation cells were incubated with a 10x serial dilution starting at 10µg/ml AZN-D1, washed and cultured for 1 hour with (glyco)-dendrimers at 37^o^C. Liposomes with LeY were taken along as positive control for AZN-D1 blocking assays. For DC-SIGN block using mannan moDC were pre-incubated for 30 minutes with a 10x serial dilution starting at 100µg/ml, followed by co-incubation with 0,01µM (glyco)-dendrimers.

### Imaging microscopy

MoDC or primary LC were pulsed for 3 hours with glyco-dendrimers at 37^ o^C, as described above. Cells were transferred to ice and stained with anti-human CD1a-biotin (clone HI149; Biolegend) for 30 minutes, washed and subsequently stained using streptavidin-AF555 (Invitrogen) for 30 minutes on ice. Next, cells were washed in ice cold PBS and fixed with 4% PFA for 20 minutes on ice prior to nuclei stain using DAPI for 10 minutes at RT. Cells were mounted on slides using MoWIOL. Z-stack images were taken with the Leica DM6000 at 63x magnification and images were analyzed using Imaris Software.

### Antigen presentation assay

APC were seeded in 96-wells plates at a concentration of 2x10^5^/ml and pulsed with (glyco)-dendrimers or DMSO vehicle control in complete medium for 3 hours at 37^o^C. MoDC were pulsed in presence or absence of 10µg/ml MPLA (Invivogen) and primary LCs of 20µg/ml Poly I:C (Invivogen). Pulsed APC were washed and co-cultured with the gp100_280-288_ specific T cell clone. For T cell degranulation, moDC were washed two times at 900rpm to remove free products and co-cultured with gp100 T cells in a 3:1 effector to target ratio for 45 minutes at 37^o^C. To measure degranulation cells were stained with a FVD, anti-human CD8 BV421 (clone RPA-T8, BD), CD107a (clone H4A3, Biolegend) and CD107b Fitc (clone H4B4, Biolegend).

For IFNγ production by T cells, moDC were pulsed for 30 minutes and LC for 3 hours at 37^o^C and co-cultured in a 1:5 effector to target ratio for 16-21 hours. IFNγ production was measured in supernatant using human cytokine ELISA (IFNγ Ready-Set-Go kit, eBioscience) according to manufacturer's protocol. Incubation with a short peptide containing the HLA-A2 minimal epitope was used to set maximum activation levels per experiment.

### *In situ* human skin DC targeting

Human skin explants (obtained as described above) were prepared by cleaning with PBS supplemented with gentamycin. Products were diluted in serum free IMDM prior to injection. Insulin needles were used to inject 20µl/biopsy i.d. at 66pmol/ml so a small blister appeared. A punch biopsy (8mm; Microtec) surrounding the blister was taken and 8 biopsies per condition were cultured with the epidermis facing upwards in a 48-wells-plate with 1ml IMDM complete skin medium for 48hours. Biopsies were discarded and crawl-out cells harvested and pooled per condition prior to FACS staining. To distinguish the different emigrated skin DC subsets cells were stained using the following anti-human antibodies: HLA-DR BV510, CD1a APC, CD14 AF700 (clone M5E2, Sony), CD141 BV711 (clone 1A4, Biolegend), EpCAM BV421 and FVD. For DC-SIGN and Langerin expression levels extra biopsies were taken and emigrated DCs stained with above cocktail plus anti-DC-SIGN AF488 (AZN-D1; own production) and anti-Langerin PE (10E2; Biolegend).

### Statistical analysis

Statistical analysis were performed using Graphpad Prism version 7.02 software (San Diego, CA). Statistical significance was determined using one- or two-way ANOVA followed by Tukey's, Sidak's or Dunnett's post hoc analysis as indicated per graph in figure legends. Data are represented as mean ±SD or symbol per donor. ns = not significant, *P < 0.05, **P < 0.01, ***P < 0.001, ****P < 0.0001.

## Supplementary Material

Supplementary figures and tables.

## Abbreviations

APC: antigen presenting cell
CLR: c-type lectin receptor
CRD: carbohydrate recognition domain
DC: dendritic cell
DC-SIGN: dendritic cell-specific ICAM- grabbing non-integrin
G0: generation 0
G3: generation 3
LC: Langerhans cell
Le: Lewis
MHC: major histocompatibility complex
moDC: monocyte derived DC
MR: mannose receptor
PAMAM: polyamidoamine
PRR: pathogen recognition receptor
TLR: toll-like receptor
