# Supplementary material for: Glyco-Dendrimers as Intradermal Anti-Tumor Vaccine Targeting Multiple Skin DC Subsets
Source: Theranostics. 2019 Aug 12;9(20):5797–809. doi: 10.7150/thno.35059 (PMC6735376; doi:10.7150/thno.35059)
Supplement: Supplementary file 1 — Supplementary figures and tables. [file thnov09p5797s1.pdf]

Supplementary files "Glyco-dendrimers as intradermal anti-tumor vaccine targeting multiple skin DC subsets" Duinkerken *et al.*

Supplementary data

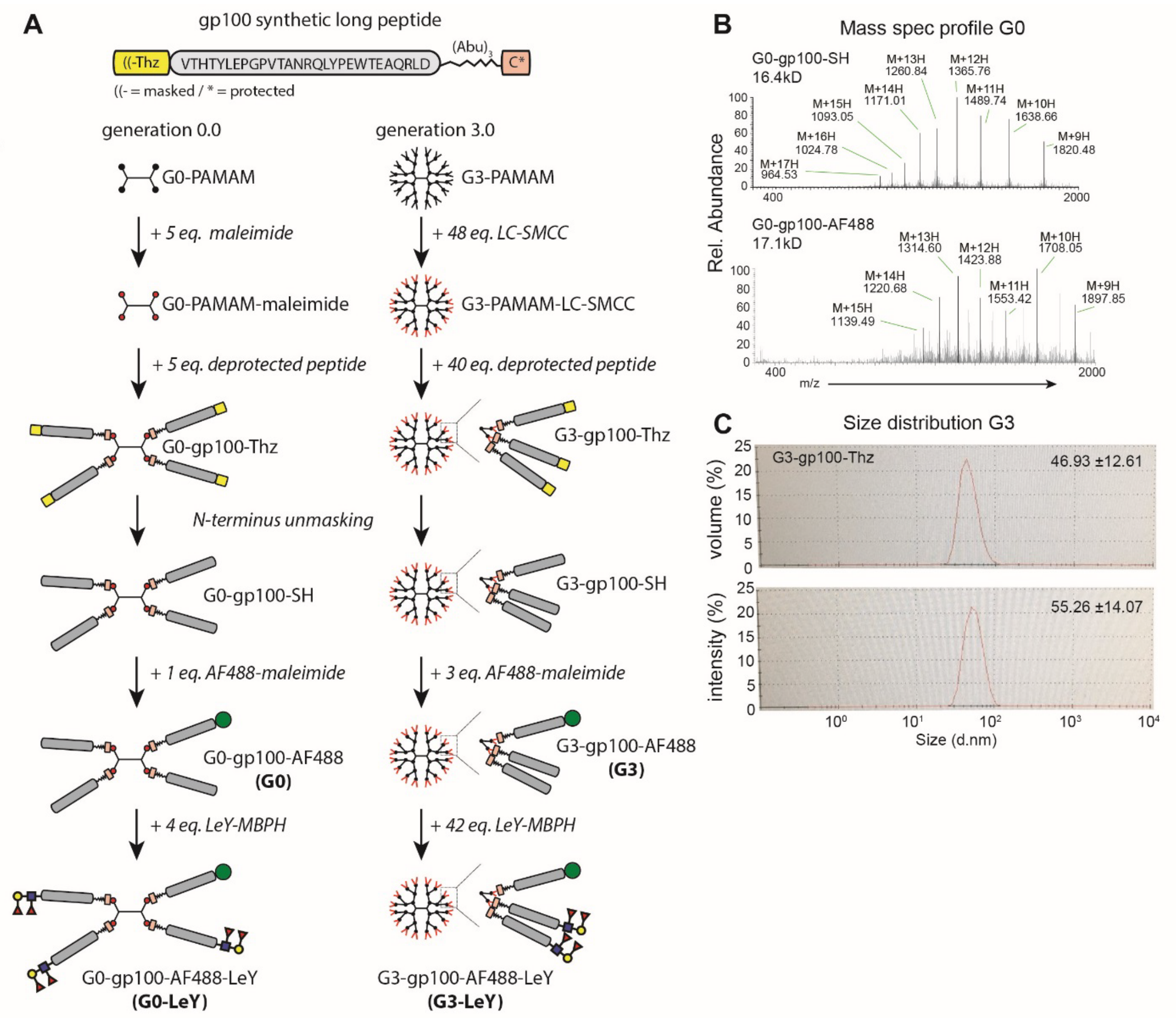

Figure S1 ***(Glyco)-dendrimer synthesis and characterization A*** *Schematic representation of (glyco)-dendrimer synthesis.* ***B*** *Mass spectrometry profile of G0-gp100-dendrimers depicting their molecular weight (MW) without (upper panel) and with AF488 (lower panel).* ***C*** *Size determination of G3-gp100-dendrimers using a dynamic and static light scattering measurement. Average particle size was determined based on volume (upper panel) and intensity (lower panel).*

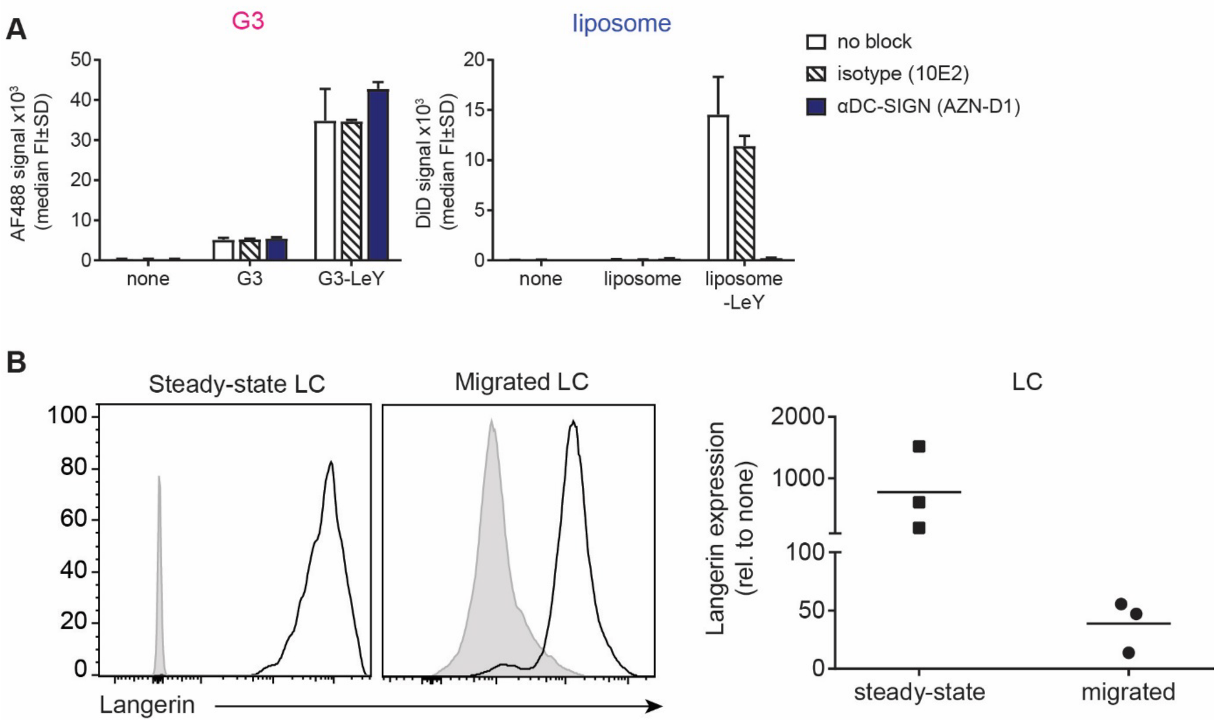

*Figure S2. **A** Binding and uptake of (glyco)-dendrimers (left panel) or (glyco)-liposomes (right panel) by moDC following pre-incubation with an anti-DC-SIGN blocking antibody or matched isotype control. **B** Langerin expression by LC isolated from human epidermal sheets using dissociation (steady-state) or two day emigration.*

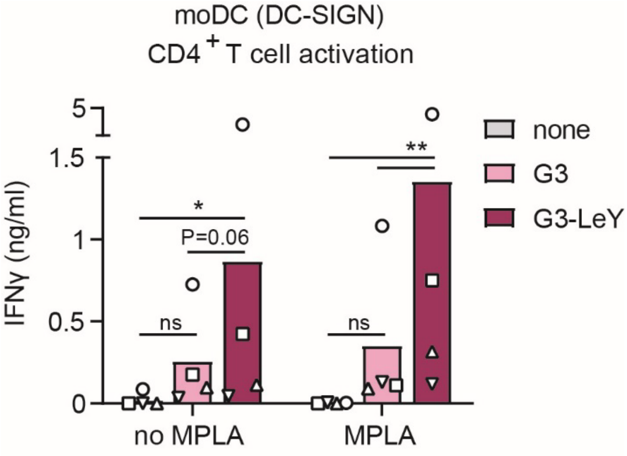

Figure S3 ***Enhanced activation of gp100 specific CD4⁺ T cells by glyco-dendrimer pulsed moDC*** *MoDC were pulsed with (glyco)-dendrimers for 30 minutes followed by an o/n co-culture with gp100 specific CD4⁺ T cells. T cell activation was evaluated by IFNγ ELISA. n=4, each symbol represents a donor. (Statistical analysis: two-way ANOVA Tukey's post hoc)*

Supplementary material and methods

MPBH functionalization of Lewis Y pentasaccharide - The bifunctional cross-linker (4-Nmaleimidophenyl) butyric acid hydrazide (MPBH) was covalently linked to the reducing end of the Lewis Y pentasaccharide via reductive amination. Briefly 1 equivalent of Lewis Y pentasaccharide (50 mg, 0.06 mmoles) was dissolved in 1 ml 20% acetic acid in DMSO containing 2.7 equivalents of MPBH (50 mg, 0.16 mmoles). 5.4 Equivalents of 2-Methylpyridine borane complex (34 mg, 0.32 mmoles) was added as reductant and the mixture was incubated at 65^{0}C for 2 hours. After 2 hours 3 ml DCM is added and vortexed thoroughly. 10 ml Diethyl ether is added to precipitate further the MPBH activated Lewis

Y pentasaccharide. After pelleting by centrifugation and pellet was washed 2 times more with diethyl ether. The activated glycan was dissolved in 0.1% TFA-MilliQ, lyophilized and subsequently purified on a preparative Ultimate 3000 HPLC system (Thermo Fisher) over a Vydac 218MS1022 C18 25x250mm column (Grace Vydac). Mass and purity were confirmed by UHPLC-MS on a Ultimate 3000 UHPLC system (Thermo Fisher) hyphenated with a LCQ-Deca XP Iontrap ESI mass spectrometer (Thermo Finnigan) using a RSLC 120 C18 Acclaim 2.2um particle 2.1 x 250 mm column and ionizing the sample in positive mode.

G0-PAMAM-Maleimide synthesis -To a solution of ethylenediamine core PAMAM Generation 0.0 dendrimer (15.4 mg, 29.81 µmoles) in 1 ml anhydrous DMSO was added to 5 equivalents of (succinimidyl 4-(N-maleimidomethyl)cyclohexane-1-carboxylate) (50 mg, 149.05 µmoles). After thoroughly vortexing 15 uL of 2,4,6-Trimethylpyridine was added in 5 aliquots of 3 µL over 30 minutes. The reaction mixture was placed on a shaker for 1 hour at room temperature. Subsequently the reaction mixture was transferred into a 50 ml falcon tube and 20 ml DCM was added. After vortexing thoroughly 25 ml Diethyl ether mixture was added to precipitate maleimide functionalized dendrimer. G0-PAMAM-maleimide was pelleted by centrifugation and washed 3 times with diethyl ether. Final product was dissolved and purified on a preparative Ultimate 3000 HPLC system (Thermo Fisher) over a Vydac 218MS1022 C18 25x250mm column (Grace Vydac). Mass and purity were confirmed by UHPLC-MS on a Ultimate 3000 UHPLC system (Thermo Fisher) hyphenated with a LCQ-Deca XP Iontrap ESI mass spectrometer (Thermo Finnigan) using a RSLC 120 C18 Acclaim 2.2um 2.1 x 250 mm column. Mass spectrometer analysis was measured in positive mode.

G3-PAMAM-LC-SMCC synthesis - To a solution of ethylenediamine core PAMAM Generation 3.0 dendrimer (16.08 mg, 2.33 µmoles) in 1 ml anhydrous DMSO was added to 48 equivalents of (succinimidyl 4-(N-maleimidomethyl)cyclohexane-1-carboxy-(6-amidocaproate)) (50 mg, 149.05 µmoles). After thoroughly vortexing 15 uL of 2,4,6-Trimethylpyridine was added in 5 aliquots of 3 µL over 30 minutes. 100 µL of 3% TFA in MilliQ was added to the reaction mixture and product was purified on a preparative Ultimate 3000 HPLC system (Thermo Fisher) over a Vydac 218MS1022 C18 25x250mm column (Grace Vydac). Fractions containing the product were pooled and lyophilized.

G0/G3-gp100-Thz synthesis - To a solution of G0-PAMAM-maleimide (3.7 mg, 2.66 µmoles) or G3-PAMAM-maleimide (5.8 mg, 0.331 µmoles) in 1 ml anhydrous DMSO either 5 (G0) or 40 (G3) equivalents of Thz-VTHTYLEPGPVTANRQLYPEWTEAQRLD-(Abu)3-C peptide (50 mg, 13.24 µmoles) were added in a 15 ml falcon tube. After thoroughly vortexing 15 uL of 2,4,6-Trimethylpyridine was added in 5 aliquots of 3 µL over 30 minutes. The reaction mixture was placed on a shaker for 3 hours at room temperature in the dark. 100 µL of 3% TFA in MilliQ was added to the reaction mixture and

product was purified on a preparative Ultimate 3000 HPLC system (Thermo Fisher) over a Vydac 218MS1022 C18 25x250mm column (Grace Vydac). G0-gp100-ThZ mass and purity were confirmed by UHPLC-MS on a Ultimate 3000 UHPLC system (Thermo Fisher) hyphenated with a LCQ-Deca XP Iontrap ESI mass spectrometer (Thermo Finnigan) using a RSLC 120 C18 Acclaim 2.2um 2.1 x 250 mm column. Mass spectrometer analysis was measured in positive mode. G3-gp100-Thz fractions containing the product were pooled and lyophilized.

G0/G3-gp100-SH - For unmasking the N-terminal thioproline lyophilized peptidic dendrimer was dissolved in 1 ml Thz-deprotection solution (NaAc buffer pH 4.5, 6M Gu.HCl, 0.1M Methoxylamine) and incubated at room temperature for 4 hours while shaking. The unmasked peptidic dendrimer G0- or G3-GP100-SH was purified over C18 500 mg SPE according to manufacturer’s protocol. Deprotected peptidic dendrimer was brought to dryness by lyophilisation.

G0/G3-Gp100-AF488/LewY construct - The G0- and G3-Gp100-SH were functionalized with either 1 (G0) or 3 (G3) equivalents of AF488-maleimide in 1 ml DMSO containing 100 mM 2,4,6-Trimethylpyridine. After 10 minutes, either 4 (G0) or 42 (G3) equivalents of Lewis Y-MPBH were added to half of the mix and the reaction was prolonged for 2 hours at room temperature on a shaker. 3 ml of DCM was added and after vortexing thoroughly 10 ml Diethyl ether was added to precipitate the labelled and glycated peptidic dendrimer. Construct was pelleted by centrifugation and washed 2 times with diethyl ether. Pellet was dissolved in MilliQ and brought to dryness by lyophilisation. Lyophilized construct was dissolved in 2 ml MilliQ-0.1% TFA and washed 6 times over 10 kD centrifugal filter to remove all excess labels and glycans. Retentate was collected and brought to dryness by lyophilisation.
